# Supplementary material for: Approaching boiling point stability of an alcohol dehydrogenase through computationally-guided enzyme engineering
Source: eLife. 2020 Mar 31;9:e54639. doi: 10.7554/eLife.54639 (PMC7164962; doi:10.7554/eLife.54639)
Supplement: Supplementary file 1. — (A) Crystallographic data. (B) Table of stabilizing mutations that disrupt activity. [file elife-54639-supp1.docx]

# Supplementary file 1

**Approaching boiling point stability of an alcohol dehydrogenase through computationally-guided enzyme engineering**

Friso S. Aalbers, Maximilian J. L. J. Fürst, Stefano Rovida, Milos Trajkovic, J. Rubén Gómez Castellanos, Sebastian Bartsch, Andreas Vogel, Andrea Mattevi, and Marco W. Fraaije

**Supplementary file 1A.** Crystallographic data of the ADHA crystal structures.

|  | Wild-type ADHA bound to NADP^+^ | Wild-type ADHA | Mutant M9 |
| --- | --- | --- | --- |
| PDB Code | 6TQ5 | 6TQ3 | 6TQ8 |
| Wavelength (Å) | 0.972640 | 0.978994 | 0.999998 |
| Resolution range (Å) | 45.54-1.60 | 44.30-2.00 | 49.16-2.50 |
| Space group | I2 | P2_1_ | P2_1_ |
| Unit cell (Å)  a, b, c (Å)  α, β, γ (°) | 76.11,113.14,124.21  90,94.0,90 | 52.52,87.25,102.92  90,101.6,90 | 51.67,86.52,102.75  90,100.6,90 |
| Unique reflections | 134154 (6625) | 61616 (4486) | 29671 (3412) |
| Multiplicity | 3.6 (3.5) | 4.8 (4.5) | 4.5 (4.8) |
| Completeness (%) | 97.4 (97.5) | 99.7 (98.6) | 96.0 (98.2) |
| Mean I/sigma (I) | 6.0 (2.0) | 14.3 (1.9) | 3.8 (1.2) |
| R-merge (%) | 15.5 (98.0) | 6.1 (61.2) | 19.3 (111.7) |
| CC_1/2_ | 0.982 (0.398) | 0.999 (0.785) | 0.979 (0.637) |
| R-work (%) | 14.1 | 19.5 | 18.9 |
| R-free (%) | 19.7 | 25.0 | 28.9 |
| Number of non-hydrogen atoms | 7849 | 7427 | 7291 |
| Protein residues | 246 | 245 | 246 |
| RMS (bonds) (Å) | 0.027 | 0.017 | 0.008 |
| RMS (angles) (°) | 2.50 | 1.84 | 1.19 |
| Ramachandran favored (%) | 97.16 | 95.57 | 93.32 |
| Ramachandran allowed (%) | 2.84 | 4.0 | 6.47 |
| Ramachandran outliers (%) | 0.00 | 0.53 | 0.21 |

**Supplementary file 1B.** Stabilizing mutations that disrupt activity.

| # | **Mutation** | **Δ*T*_m_** (°C) | *k*_obs_* (U/mg) | Possible effect(s) |
| --- | --- | --- | --- | --- |
| 1 | A155F | 14.0 ± 0.5 | < 0.01 | Blocking active site, disrupting catalysis |
| 2 | S92E | 12.0 ± 0.5 | < 0.01 | Electrostatic interaction with catalytic Lys |
| 3 | S39D | 7.8 ± 3.5 | < 0.01 | Disrupts NADPH-phosphate binding through negative charge repulsion |
| 4 | R18D | 7.0 ± 2.5 | < 0.01 | Disrupts NADPH-phosphate binding through negative charge repulsion |
| 5 | R40E | 7.0 ± 1.5 | < 0.01 | Disrupts NADPH-phosphate binding through negative charge repulsion |
| 6 | N232H | 6.5 ± 3.5 | < 0.01 | Affects / disrupts dimer interface |
| 7 | D222Q | 5.0 ± 1.5 | < 0.01 | - |
| 8 | A155Y | 4.5 ± 0 | 0.02 | Blocking active site, disrupting catalysis |
| 9 | S17D | 4.0 ± 0 | n.d. | Disrupts NADPH-phosphate binding through negative charge repulsion |
| 10 | R40P | 3.8 ± 1 | n.d. | Negatively affects NADPH-phosphate binding |
| 11 | Q44E | 3.5 ± 0.5 | < 0.01 | Disrupts NADPH-phosphate binding through negative charge repulsion |

Note that all mutations that probably disrupt NADPH-binding introduce a negative charge (aspartic or glutamic acid). Determined activities are given in units / mg / min. **k_obs_* values are averages based on 2-3 replicates, and for each average the error was smaller than 5%.
