# Supplementary material for: Approaching boiling point stability of an alcohol dehydrogenase through computationally-guided enzyme engineering
Source: eLife. 2020 Mar 31;9:e54639. doi: 10.7554/eLife.54639 (PMC7164962; doi:10.7554/eLife.54639)
Supplement: Supplementary file 2. — (A) Expression of ADHA at different temperatures. (B) Purification of SUMO-M9*. [file elife-54639-supp2.docx]

# Supplementary file 2

**Approaching boiling point stability of an alcohol dehydrogenase through computationally-guided enzyme engineering**

Friso S. Aalbers, Maximilian J. L. J. Fürst, Stefano Rovida, Milos Trajkovic, J. Rubén Gómez Castellanos, Sebastian Bartsch, Andreas Vogel, Andrea Mattevi, and Marco W. Fraaije

**
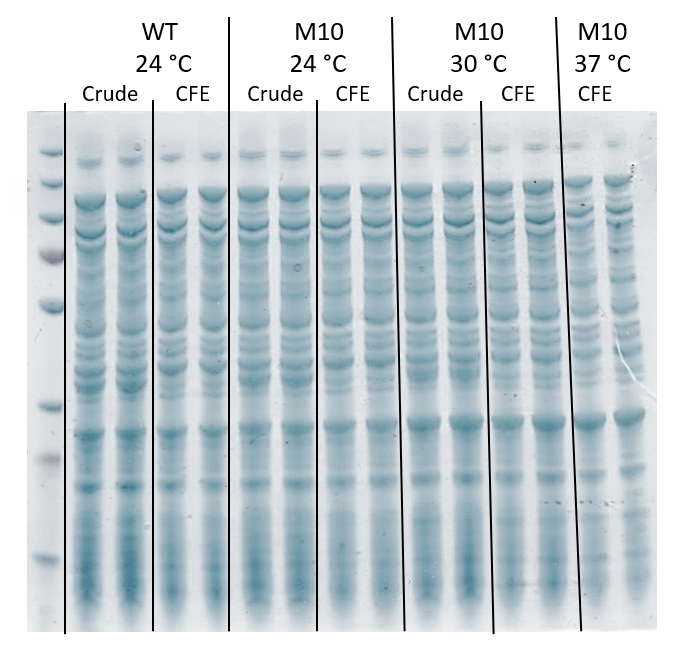

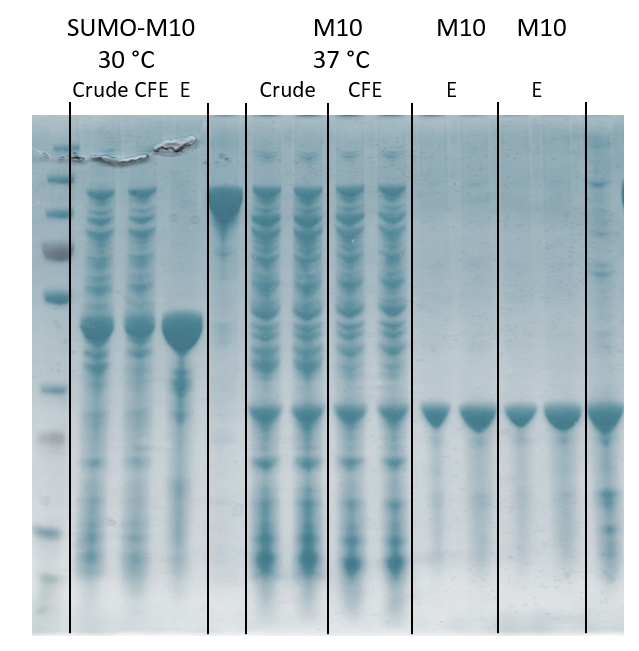
**

0

0

0

**Supplementary file 2A.** Expression comparison wild-type ADHA and M9 at different temperatures, with biological duplicates. The expression at 37 °C was 16 hours (overnight), whereas the expression at 30 °C and 24 °C were for 38 hours. Protein ladder (first lane on the left) is pre-stained PageRuler (ThermoScientific, MA USA). The black arrow indicated the position of the ADHA on the gel. Crude = crude extract, after sonication and before centrifugation. CFE = cell-free extract, after spinning down cell-debris. E = elute, fraction collected after addition of imidazole, during IMAC purification.


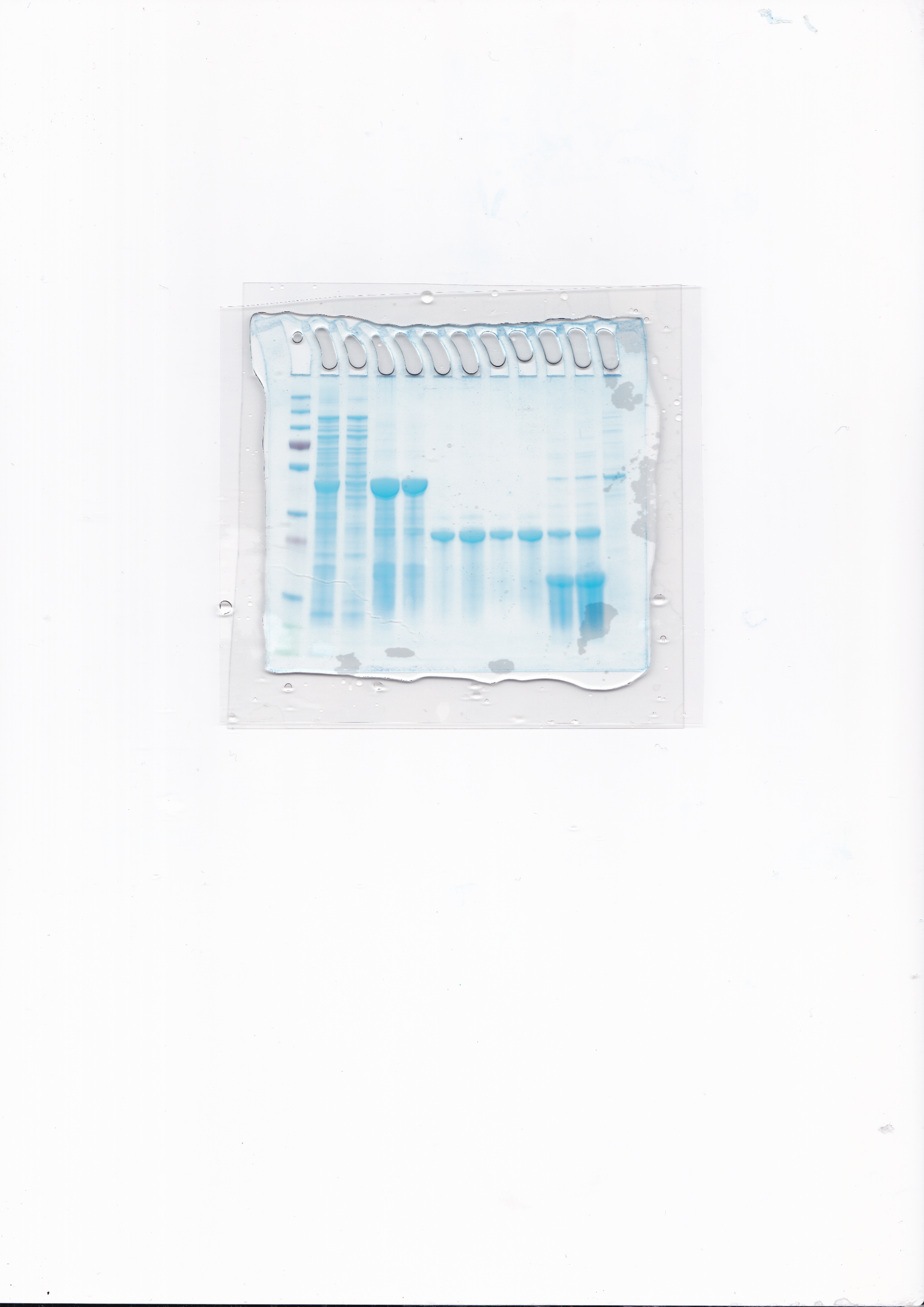


1 2 3 4 5 6 7 8 9 10 11

0

0

**Supplementary file 2B.** Purification of His-SUMO-M9*. The left arrow indicates the size of SUMO-M9*, the arrow in the right side indicates the size of M9* after SUMO cleavage. 1. Ladder, 2. Cell-free extract, 3. Flow-through, 4. First elute, 5. Second elute, 6. First flow-through after SUMO cleavage (low concentration), 7. First flow-through after SUMO cleavage (high concentration), 8. Second flow-through after SUMO cleavage (low concentration), 9. Second flow-through after SUMO cleavage (high concentration), 10. and 11. Elute with imidazole after SUMO cleavage.
