# Supplementary material for: Approaching boiling point stability of an alcohol dehydrogenase through computationally-guided enzyme engineering
Source: eLife. 2020 Mar 31;9:e54639. doi: 10.7554/eLife.54639 (PMC7164962; doi:10.7554/eLife.54639)
Supplement: Supplementary file 3. — (A) Correlation of predicted values and experimental melting point data. (B) Graph of correlation. [file elife-54639-supp3.docx]

# Supplementary file 3

**Approaching boiling point stability of an alcohol dehydrogenase through computationally-guided enzyme engineering**

Friso S. Aalbers, Maximilian J. L. J. Fürst, Stefano Rovida, Milos Trajkovic, J. Rubén Gómez Castellanos, Sebastian Bartsch, Andreas Vogel, Andrea Mattevi, and Marco W. Fraaije

**Supplementary file 3A.** Predicted values (in Gibbs free energy ΔΔ*G*, kJ/mol) of hits and correlation to experimental data (apparent melting temperatures).

|  | Apparent melting T | Number of values | Average (ΔΔ*G* (kJ)) | Median  (ΔΔ*G* (kJ)) | Standard deviation |
| --- | --- | --- | --- | --- | --- |
| FoldX | Selected (Δ*T*_m_ ≥ 3 ˚C) | 5 | -35 | -36 | ± 5 |
| FoldX | Δ*T*_m_ ≥ 3 ˚C | 10 | -38 | -36 | ± 14 |
| FoldX | Δ*T*_m_ ≥ 2 ˚C | 16 | -34 | -33 | ± 17 |
| FoldX | Δ*T*_m_ < 2 ˚C | 47 | -22 | -19 | ± 14 |
| FoldX | Δ*T*_m_ ≤ 0 ˚C | 29 | -21 | -18 | ± 12 |
| Rosettaddg | Selected (Δ*T*_m_ ≥ 3 ˚C) | 9 | -17 | -18 | ± 7 |
| Rosettaddg | Δ*T*_m_ ≥ 3 ˚C | 25 | -15 | -12 | ± 9 |
| Rosettaddg | Δ*T*_m_ ≥ 2 ˚C | 43 | -16 | -13 | ± 11 |
| Rosettaddg | Δ*T*_m_ < 2 ˚C | 108 | -14 | -11 | ± 10 |
| Rosettaddg | Δ*T*_m_ ≤ 0 ˚C | 74 | -14 | -11 | ± 9 |

As with **Supplementary file 3B,** the FoldX values show better correlation with the experimental data. The lower the number of values for a particular correlation, the less valuable the correlation, due to low sample size.


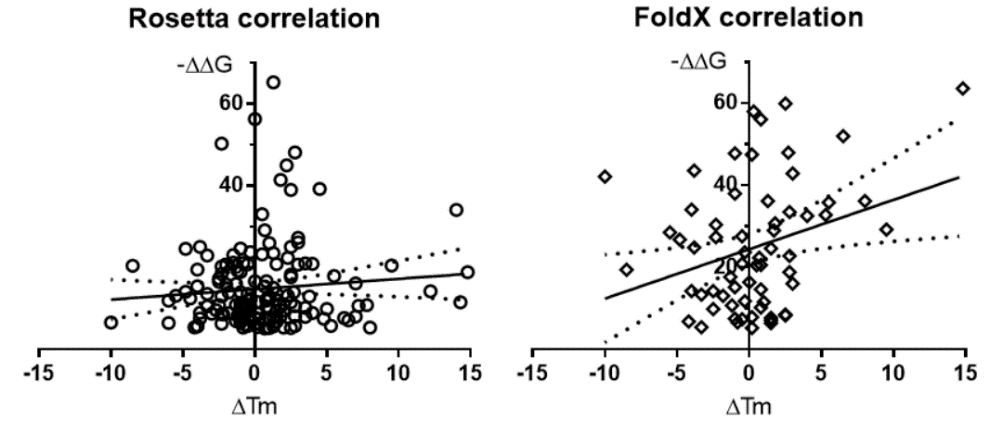


**Supplementary file 3B.** Calculated values compared to observed melting temperatures, fitted with linear regression in GraphPad prism 6.07. The graph depicts the slope from the linear regression, with the dotted lines representing the 95% confidence intervals of the slopes. Rosetta (left pane, circles): Pearson correlation of r = 0.09; no significant correlation (P = 0.25) between apparent *T*_m_ measurements and calculated stabilization (ΔΔ*G*^Fold^, more negative is more stable) FoldX (right pane, diamonds): Pearson correlation of r = 0.3; significant correlation (P < 0.02).
