# Supplementary material for: Approaching boiling point stability of an alcohol dehydrogenase through computationally-guided enzyme engineering
Source: eLife. 2020 Mar 31;9:e54639. doi: 10.7554/eLife.54639 (PMC7164962; doi:10.7554/eLife.54639)

# Supplementary file 4

**Approaching boiling point stability of an alcohol dehydrogenase through computationally-guided enzyme engineering**

Friso S. Aalbers, Maximilian J. L. J. Fürst, Stefano Rovida, Milos Trajkovic, J. Rubén Gómez Castellanos, Sebastian Bartsch, Andreas Vogel, Andrea Mattevi, and Marco W. Fraaije

**Supplementary file 4A. Settings for GC and HPLC**

**GC:**

Column: Chirasil-DEX CB column (25 m × 0.25 mm)

Injector temperature: 250°C

Detector temperature: 250°C

Column temperature: 110°C kept for 2 min, 2°C/min increased to 126°C, kept for 5 min, and

then 2°C/min increased to 160°C

Injection volume: 1 μL

Split ratio: 50

Retention times: ketone (8.363 min), (*R*)-alcohol (12.500 min), (*S*)-alcohol (12.563 min)

**HPLC:**

Column: Chiralcel OD-H column (4.6 mm × 250 mm)

Detector wavelength: 254 nm

Mobile phase: n-Heptane/isopropanol (95/5)

Flow rate: 1 mL/min

Injection volume: 10 μL

Retention times: ketone (5.140 min), (*R*)-alcohol (8.905 min), (*S*)-alcohol (8.179 min).

**Supplementary file 4B. GC chromatograms for ethyl-4-chloro-3-oxobutanoate (COBA)**

**Order of chromatograms:** **CTRL** – substrate in the buffer, **PTDH** – substrate with everything except ADH, **WT** – reaction with ADHA wildtype, **M9F** – reaction with ADHA M9*, **R-CHBE** – Ethyl (R)-(+)-4-chloro-3-hydroxybutyrate commercial standard, **S-CHBE** – Ethyl (S)-(−)-4-chloro-3-hydroxybutyratecommercial standard.

**Overlaid GC chromatograms for ethyl-4-chloro-3-oxobutanoate (COBA)** – WT (green), M9* (purple), R-CHBE (red) and S-CHBE (blue).

**Supplementary file 4C. HPLC chromatograms for 4-chloroacetophenone (4-CAP)**

**Order of chromatograms:** **A** – substrate in the buffer, **B** – substrate with everything except ADH, **C** – reaction with ADHA wildtype, **D** – reaction with ADH M9*, **E** – (R)-1-(4-Chlorophenyl)ethanol standard, **F** – (S)-1-(4-Chlorophenyl)ethanol standard, **G** – Racemic 1-(4-Chlorophenyl)ethanol standard.

**A** – Substrate in the buffer (control)


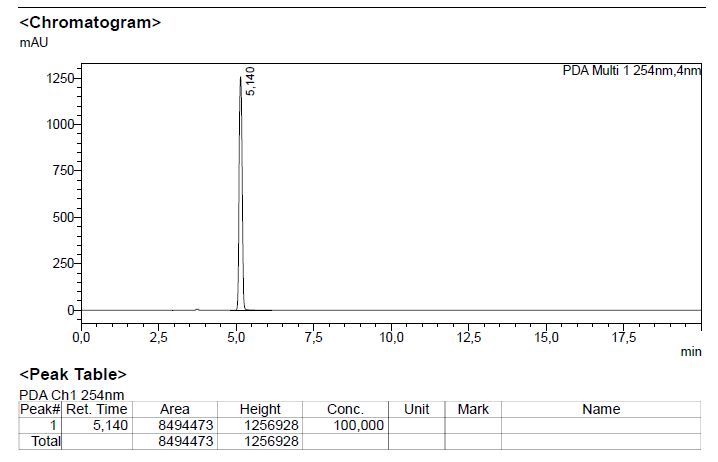


**B** – Substrate with everything except ADH

**
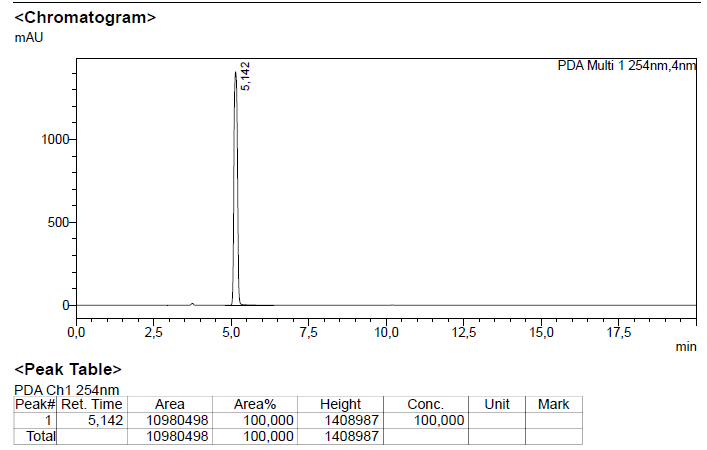
**

**C** – Reaction with ADHA wildtype


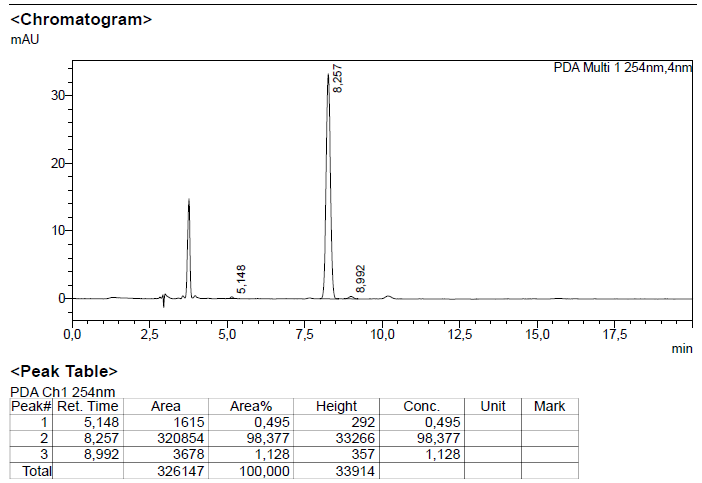


**D** – Reaction with ADH M9*


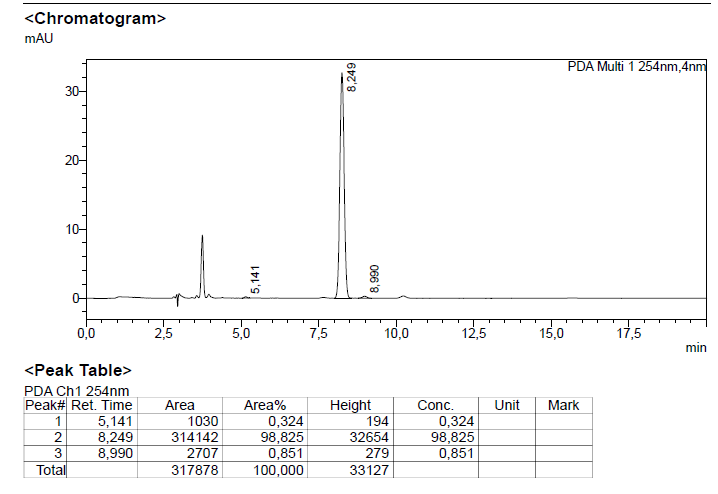


**E** – (R)-1-(4-Chlorophenyl)ethanol standard


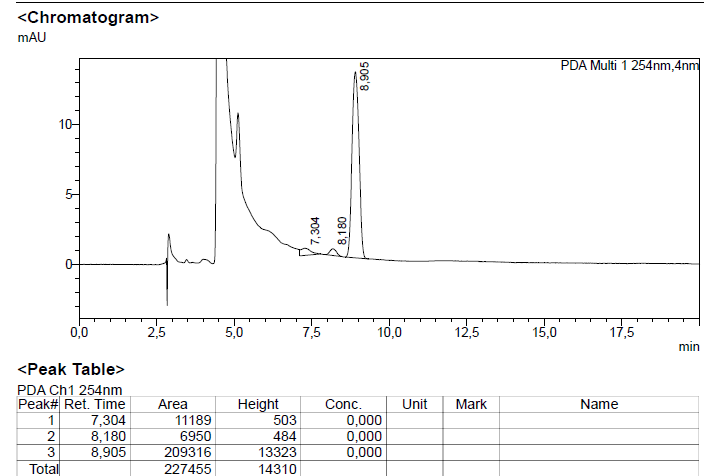


**F** – (S)-1-(4-Chlorophenyl)ethanol standard
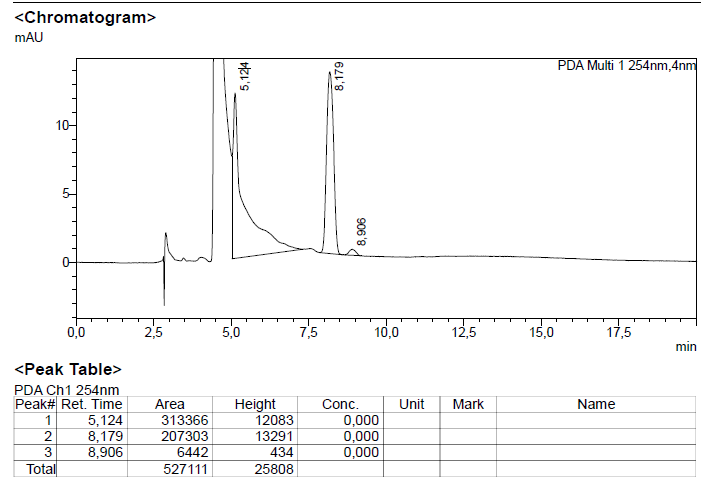


**G** – Racemic 1-(4-chloro)-phenylethanol standard


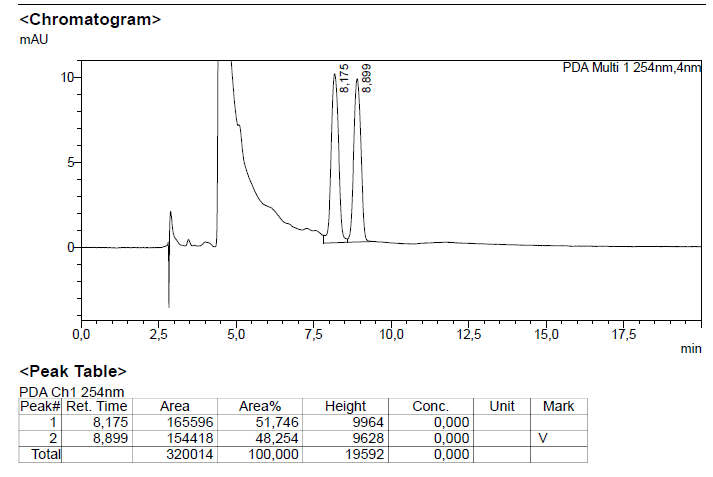

Supplement: Supplementary file 4. — (A) Settings for GC and HPLC. (B) Chromatograms GC. (C) Chromatograms HPLC. [file elife-54639-supp4.docx]
